# Safety and Effectiveness of two treatment regimes with tranexamic acid to minimize inflammatory response in elective cardiopulmonary bypass patients: a randomized double-blind, dose-dependent, phase IV clinical trial

**DOI:** 10.1186/1749-8090-6-138

**Published:** 2011-10-14

**Authors:** Juan J Jiménez, José L Iribarren, Maitane Brouard, Domingo Hernández, Salomé Palmero, Alejandro Jiménez, Leonardo Lorente, Patricia Machado, Juan M Borreguero, José M Raya, Beatriz Martín, Rosalía Pérez, Rafael Martínez, María L Mora

**Affiliations:** 1 Critical Care Department. Hospital Universitario de Canarias. Ofra s/n, La Cuesta. 38320-La Laguna. España; 2 Nephrology Department. Hospital Universitario Carlos Haya, 29010-Málaga. España; 3 Mixed Research Unit. Hospital Universitario de Canarias. Ofra s/n, La Cuesta. 38320-La Laguna. España; 4 Hematology Laboratory. Hospital Universitario de Canarias. Ofra s/n, La Cuesta. 38320-La Laguna. España; 5 Biochemical laboratory. Hospital Universitario de Canarias. Ofra s/n, La Cuesta. 38320-La Laguna. España; 6 Cardiac Surgery Department. Hospital Universitario de Canarias. Ofra s/n, La Cuesta. 38320-La Laguna. Tenerife. España

**Keywords:** Cardiac surgery, Cardiopulmonary bypass, Fibrinolysis, Tranexamic acid, Inflammatory response, Bleeding

## Abstract

**Background:**

In cardiopulmonary bypass (CPB) patients, fibrinolysis may enhance postoperative inflammatory response. We aimed to determine whether an additional postoperative dose of antifibrinolytic tranexamic acid (TA) reduced CPB-mediated inflammatory response (IR).

**Methods:**

We performed a randomized, double-blind, dose-dependent, parallel-groups study of elective CPB patients receiving TA. Patients were randomly assigned to either the single-dose group (40 mg/Kg TA before CPB and placebo after CPB) or the double-dose group (40 mg/Kg TA before and after CPB).

**Results:**

160 patients were included, 80 in each group. The incident rate of IR was significantly lower in the double-dose-group TA2 (7.5% vs. 18.8% in the single-dose group TA1; *P *= 0.030). After adjusting for hypertension, total protamine dose and temperature after CPB, TA2 showed a lower risk of IR compared with TA1 [OR: 0.29 (95% CI: 0.10-0.83), (*P *= 0.013)]. Relative risk for IR was 2.5 for TA1 (95% CI: 1.02 to 6.12). The double-dose group had significantly lower chest tube bleeding at 24 hours [671 (95% CI 549-793 vs. 826 (95% CI 704-949) mL; *P *= 0.01 corrected-*P *significant] and lower D-dimer levels at 24 hours [489 (95% CI 437-540) vs. 621(95% CI: 563-679) ng/mL; *P *= 0.01 corrected-*P *significant]. TA2 required lower levels of norepinephrine at 24 h [0.06 (95% CI: 0.03-0.09) vs. 0.20(95 CI: 0.05-0.35) after adjusting for dobutamine [F = 6.6; *P *= 0.014 corrected-*P *significant].

We found a significant direct relationship between IL-6 and temperature (rho = 0.26; *P *< 0.01), D-dimer (rho = 0.24; *P *< 0.01), norepinephrine (rho = 0.33; *P *< 0.01), troponin I (rho = 0.37; *P *< 0.01), Creatine-Kinase (rho = 0.37; *P *< 0.01), Creatine Kinase-MB (rho = 0.33; *P *< 0.01) and lactic acid (rho = 0.46; *P *< 0.01) at ICU arrival. Two patients (1.3%) had seizure, 3 patients (1.9%) had stroke, 14 (8.8%) had acute kidney failure, 7 (4.4%) needed dialysis, 3 (1.9%) suffered myocardial infarction and 9 (5.6%) patients died. We found no significant differences between groups regarding these events.

**Conclusions:**

Prolonged inhibition of fibrinolysis, using an additional postoperative dose of tranexamic acid reduces inflammatory response and postoperative bleeding (but not transfusion requirements) in CPB patients. A question which remains unanswered is whether the dose used was ideal in terms of safety, but not in terms of effectiveness.

**Current Controlled Trials number:**

ISRCTN: ISRCTN84413719

## Background

Excessive bleeding and inflammatory response (IR) after cardiopulmonary bypass (CPB) are common complications of cardiac surgery [1]. Although tranexamic acid (TA), a lysine analog competitive inhibitor of plasmin and plasminogen [2], is effective in reducing bleeding after cardiac surgery, its anti-inflammatory effect in fibrinolysis blockade has been less studied.

In vitro studies suggest that the exposure of D-dimer fragments to monocytes initiates the synthesis and release of IL-6 [3]. Excessive plasmin activity and/or D-dimer formation may play an important role in proinflammatory cytokine and cellular response activation during CPB [4]. In patients undergoing elective CPB, prophylactic blockade of fibrinolysis decreases molecular expression of IR [5]. This IR may contribute to postoperative complications, including myocardial dysfunction, respiratory failure, renal and neurologic dysfunction, bleeding disorders, altered liver function, and ultimately, multiple organ failure (MOF) [4]. In a previous study using TA before and after CPB we observed reduced bleeding and IR [6]. These results were encouraging but left unanswered the question of whether the postoperative dose, coinciding with greatest fibrinolytic activation, was determinant in decreasing postoperative IR and bleeding. Nevertheless newer data indicate that, in a dose-dependent fashion, TA is associated with an increase of adverse events, particularly the observation of seizures [7]. The incidence of this adverse effect varies from 2.7% to 4.6% in any major surgical procedure, independently of dosing schedule [8-10]. For this reason, we also focused on the safety profile of TA dosing schedule as a part of the study.

## Methods

The study was approved by our university hospital ethics committee and conducted according to the Helsinki Declaration. We report this trial in accordance with the CONSORT statement revised recommendations [11].

From December 2005 to January 2007, we performed a randomized, double-blind, dose-dependent parallel-groups study with 160 consecutive adult patients undergoing elective CPB, in a 24-bed intensive care unit (ICU) at a university hospital.

Exclusion criteria included: no informed consent, age < 18 years, emergencies, off-pump cardiac surgery, chronic coagulopathy (prothrombin time [PT] <50% or international normalized ratio (INR) >2 and platelets <50,000/mm^3 ^or aggregation dysfunction), renal failure (creatinine >2 mg/dL), gross hematuria, TA hypersensibility, chronic hepatopathy (Child-B or higher), immunosuppression, endocarditis and post-operative sepsis within 24 h.

Before CPB, all participants had normal bleeding time, platelet collagen/epinephrine and collagen/ADP closure time, PT, activated partial thromboplastin time, and thrombin time. No patients received anti-inflammatory or immunosuppressive agents during 5 days before and 24 h post-CPB.

After obtaining written informed consent, the surgeon requested treatment group assignment immediately before CPB.

### TA dosing schedule

Dosing schedules for prophylactic TA varies greatly [12,13]. In our center, the TA dosing schedule is an initial bolus (25 mg/Kg) before CPB and another (25 mg/Kg) after completion of CPB. Our first study dealing with TA versus placebo was carried out using this schedule, which has demonstrated its effectiveness in relation to bleeding and IR [6]. Given the half-life of TA (80 min), the initial dose inhibits intra-operative fibrinolysis and bleeding. A loading dose higher than 30 mg/Kg assures a 98-100% reduction of tissue activator activity [14]. Thus the dose schedule used in our study ensured complete inhibition of fibrinolysis during and early postoperative period after CPB, although no pharmacokinetic information was available. With the second dose we wished to investigate the possible benefit of postoperative inhibition of fibrinolysis on IR.

The patients were randomly assigned, by an independent pharmacist according to a computer generated randomization list allocated in the Pharmacology Department, to receive disguised coded infusions of either TA 40 mg/Kg before CPB and placebo after CPB (**TA1**) or TA 40 mg/Kg before and after CPB (**TA2**) after heparin reversal. The code was revealed once recruitment, data collection, and laboratory analyses were completed.

### Anesthetic procedures

Anesthetic procedures were standardized and consisted of an opioid-based anesthetic supplemented with volatile anesthetic and muscle relaxants. All interventions were performed by the same surgical team with wide experience in these surgical interventions. All patients were preoperatively monitored with a pulmonary artery continuous thermodilution catheter (Edwards Lifesciences LLC, Irvine, CA, USA). Neither heparin-coated circuits nor leukocyte filters were used. The extracorporeal circuit consisted of a hardshell membrane oxygenator (Optima XP; Cobe, Denver, CO, USA, or Quantum Lifestream International, Inc., Woodlands, TX, USA), a Tygon™ (Dideco s.r.l., Mirandola, Italy) extracorporeal circuit, and a Medtronic™ Biopump (Medtronic, Inc., Minneapolis, MN, USA) centrifugal pump. Below hypothermic temperatures of 28°C to 30°C, the pump flow was adjusted to maintain a mean arterial pressure of greater than 60 mm Hg and a flow index of 2.2 L/minute per square meter. Myocardial protection was achieved using antegrade, cold, St. Thomas 4:1 sanguineous cardioplegia. The circuit was primed with 30 mg of heparin followed by an initial dose of 3 mg/kg and further doses when necessary to achieve and maintain an activated clotting time of 480 seconds. To reverse the effect of heparin, protamine was used based on blood heparin levels measured by Hepcon^® ^(Medtronic, Inc.). A blood salvage device (Cobe BRAT2™, Cobe Cardiovascular Inc.TX. USA) was used in all patients. The transfusion trigger was a hemoglobin threshold of less than 8 g/dL, PT of less than 50%, and platelets of less than 50,000/mm3. Fluid management was carried out to achieve 8 to 12 mm Hg of central venous pressure or 12 to 15 mm Hg of pulmonary artery occlusion pressure at zero positive end-expiratory pressure by infusions of crystalloids and colloids. Following our routine practice, we used Ringer Lactate or Saline 0.9% in the intra-operative period. After patient admission to ICU, crystalloids were mainly used and when colloids were infused we most commonly used HES 130/0.4 (Voluven ^®^), not exceeding 1000 ml in 24 hours.

Catecholamine support, when necessary, was used as follows: Norepinephrine was titrated to achieve a mean arterial pressure of greater or equal to 70 mm Hg, and dobutamine was titrated to achieve a cardiac index of greater or equal to2.5 L/minute per square meter. Amines were tapered off in steps of 0.02 and 1 μg/kg per minute, respectively.

### Concurrent validation

We validated the clinical criteria of IR and found significant differences in evolutionary levels of IL-6 in patients who developed IR versus those without.

## Data collection

Demographic variables, comorbidity, perioperative clinical data, and postoperative IR, mechanical ventilation time, ICU and hospital stay, and mortality, were recorded. Core body temperature, laboratory data (hematology, inflammation, coagulation, and fibrinolysis), and hemodynamic parameters were recorded before intervention (baseline), on ICU admission after surgery (0 h), and at 4 h and 24 h post-CPB, once hemodynamic stability was confirmed. IR was clinically defined as core body temperature >38°C (100.4°F), systemic vascular resistance index <1,600 dyne · sec/cm^5 ^per m^2^, and cardiac index >3.5 L/minute per m^2 ^at 4 h, as we reported in a previous study [6]. We also recorded blood loss (chest-tube drainage and hemoderivatives) at the above time points and on chest tubes removal. In cases of reintervention due to bleeding, the post-hoc classification of bleeding into "surgical" and "non-surgical" was applied to distinguish between bleeding due to mechanical-surgical causes (where pharmacological measures are not effective) and coagulopathy (which TA was expected to reduce). Surgical risk was calculated by Euroscore and Parsonnet score.

Cerebrovascular events included seizure and stroke. Acute kidney injury was assessed applying RIFLE [15] criteria (>100% creatinine increase, using preoperative and highest creatinine concentration during the first week after surgery); renal failure was defined as dysfunction requiring dialysis. Myocardial infarction was considered as either new Q waves or new, persistent ST-segment or T-wave changes. Indication for re-operation was determined by clinical judgment and blood loss as >200 mL/h in three consecutive hours. Mortality was defined as death within 30 days of CPB, recorded in medical history or telephone contact with the surgeon responsible or family. These adverse effects were included as a composite variable.

## Cytokine levels

IL-6 (normal range: <5.9 pg/mL; intra-assay variation: 4.5%) was measured by automatic immunoenzyme assay (IMMULITE ONE™; Diagnostic Products Corporation).

## Coagulation and fibrinolysis determination

D-dimer (normal range: < 300 ng/mL; intra-assay variation: 3%) was measured by immunoturbidimetric test (D-dimer PLUS; Dade Behring).

## Statistical analysis

### Sample size

In relation to the power of the study, prior experimental information available is as follows: the proportion of IR in patients receiving 30 mg/kg in two doses was 16.6% [6]; however, we had no prior information on the expected incidence rate of IR with 80 mg/kg. In the present study, we hypothesized that the double dose of TA would reduce the inflammatory response. For expected incidences of 16.6% in the TA1 group (40-0 mg/Kg) and 3% in the TA2 group, with 80% power and 5% type one error (one-tailed), 58 patients per group were needed. Assuming 28% drop-out, 80 patients were required in each treatment group.

For the concurrent validation of the clinical criteria of IR, we used mixed ANOVA with repeated measures of evolutionary levels of IL-6 (baseline, at ICU arrival, and at 4 h postoperatively), after log-transformation. The intergroup variable was IR (yes or no). After applying the Bonferroni correction, differences with a p value of <0.018 were considered significant.

Comparisons between groups (TA1 versus TA2) were performed per protocol analyses, using Pearson's χ^2 ^test or Fischer's exact test for categorical variables, and Student's t-test or Mann-Whitney's U test for continuous variables. Assumption of normality of the TA groups was tested with the Kolmogorov-Smirnov test, and homoscedasticity was tested with the Levene test. When any adjustment or multiple comparisons were necessary on applying analysis of covariance or mixed ANOVA, the variables were previously log-transformed to ensure normal distribution and homoscedasticity.

All preoperative variables showing a *P *value < 0.15 in the bivariate analysis (hypertension, post-CPB temperature, total protamine dose) were entered in a multivariate binary logistic regression analysis, for categorical primary end-point outcome (IR).

Mixed ANOVA was used to compare means between groups in clinical signs (bleeding) and laboratory parameters (D-dimer, creatine kinase and troponin I) at baseline, 4 h and 24 h after surgery. For bleeding and D-dimer, correction for multiple variables was not carried out in the omnibus test because of the *a priori *known unidirectional nature of the effects of TA on these parameters. However, any isolated values in repeated measures were considered significant when *P *< 0.018, after applying Bonferroni correction. In the case of 24 h bleeding, this was adjusted for surgery-related bleeding.

To test the effect of TA on 24 h postoperative norepinephrine requirements, the statistical analysis included adjustment for the use of dobutamine. After applying Bonferroni correction, differences with a *P *value of < 0.018 were considered significant.

Bivariate associations between IL-6 and D-dimer, chest-tube bleeding, norepinephrine dose, temperature, troponin I (TnI), creatine kinase (CK), MB isoenzyme creatine kinase (CK-MB) and blood pressure were assessed using Spearman's rho coefficient.

Qualitative variables are expressed as frequencies and percentages and quantitative variables as mean and 95% confidence interval (CI), or median and interquartile range. *P *values of < 0.05 were considered significant. SPSS 15.0.1 (SPSS Inc. Chicago, IL. USA) and Statistica 8.0 (StatSoft, Tulsa, OK) were used.

## Results

**Figure 1:**
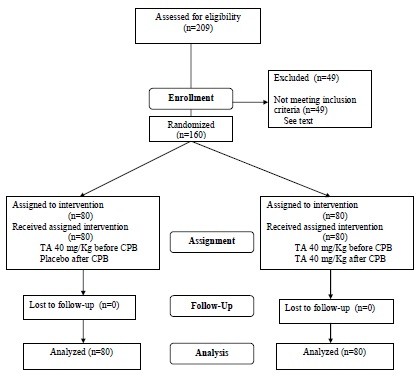
**Randomized control trial flow diagram**.

From December 2005 to January 2007, we recruited 209 consecutive CPB patients; 49 were excluded (20 off-pump, 4 coagulation disorders, 11 emergencies, 6 endocarditis, 4 hemodialysis, and 4 immunosuppressed). Thus we studied 160 patients, 80 receiving single-dose TA before CPB and 80 receiving TA before and after CPB. Figure 1 shows a flow chart of patients enrolled.

Demographic variables, comorbidity, medical treatment, preoperative biochemical data, and surgical procedures were similar in the two groups, except for post-CPB temperature and total protamine dose (Table 1).

**Table 1:** Baseline characteristics of treatment groups.

	Tranexamic acid		*P*
	TA1 group (40-0 mg/Kg) (n = 80)	TA2 group 40-40 mg/Kg (n = 80)	
**Demographics**			
Age (years)	65 (62-69)	68 (65-70)	0.24
Male sex, n (%)	54 (67.5)	49 (61.3)	0.41
Parsonnet	9.2 (7.2-11.2)	10.5 (8.4-12.5)	0.30
Euroscore^a^	4 (3-6)	4 (3-7)	0.38
Logistic Euroscore^a^	3.25(1.83-5.53)	3.27(1.75-6.68)	0.84
Body mass index (Kg/m^2^)	28.2 (27.4-29)	27.7 (26.6-28.8)	0.50
**Comorbidity**			
Hypertension, n (%)	43 (53.8)	53 (66.3)	0.11
Diabetes, n (%)	36 (45)	28 (35)	0.42
**Medical treatment**			
Angiotensin-converting enzyme inhibitors, n (%)	28 (35)	22 (27.5)	0.31
**Preoperative parameters**			
Platelet count (× 10^3 ^ml^-1^)	247 (227-267)	262 (239-285)	0.48
D-dimer (ng/ml)	347 (217-476)	287 (221-353)	0.95
International normalized ratio	1.04 (1-1.07)	1.05 (1.01-1.09)	0.28
**Cardiac intervention**			
Coronary, n (%)	45 (56.3)	39 (48.8)	0.51
Valvular, n (%)	25 (31.3)	31 (38.8)	
Both, n (%)	9 (11.3)	7 (8.8)	
Other, n (%)^b^	1 (1.3)	3 (3.8)	
Second intervention, n (%)	4 (5)	3 (3.8)	0.51
**Surgical data**			
Temperature during cardiopulmonary bypass (°C)	32.3 (32-32.5)	32.3 (32.1-32.5)	0.78
Aortic clamp time (min)	53 (49-58)	51 (46-57)	0.61
Cardiopulmonary bypass time(min)	88 (82-94)	84 (77-92)	0.45
Temperature after cardiopulmonary bypass (°C)	35.5 (35.3-35.6)	35.7 (35.5-35.8)	0.03
Total heparin dose (UI/Kg)	391 (371-412)	400 (379-421)	0.56
Total protamine dose (mg/Kg)	2.7 (2.6-2.8)	2.9 (2.8-3.1)	0.04
Heparine/protamine	1.45 (1.37-1.53)	1.39 (1.33-1.44)	0.29
Blood salvage (ml)	724 (663-784)	703 (636-767)	0.64

Values expressed as mean and 95% confidence interval; frequencies and percentages; ^a^median and percentiles.

^b^Inter-atrial communication.

**Figure 2:**
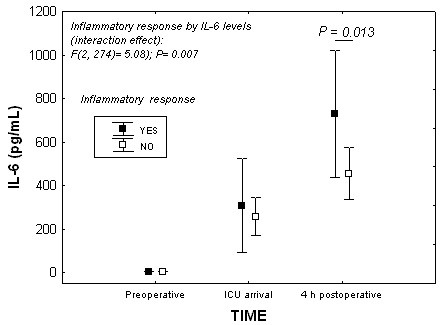
**Mixed ANOVA showing the relationship between IR and IL-6 during the follow up**.

Patients developing IR showed significantly higher levels of IL-6 at 4 hours postoperatively than those without IR, [F (2, 274) = 5.08; *P *= 0.013], (Figure 2).

The incident rate of IR was significantly lower in TA2 (7.5%) than in TA1 (18.8%) (*P *= 0.030). TA2 showed OR 0.29 (95%CI: 0.10-0.83), (*P *= 0.013) after adjusting for hypertension, total protamine dose and temperature after CPB. Compared with TA2, the relative risk of IR was 2.5 for TA1 (95% CI 1.02-6.12). The absolute risk difference was 11.3%. The number needed to treat with TA2 to reduce IR was 9 patients (95% CI: 5-107 patients).

**Figure 3:**
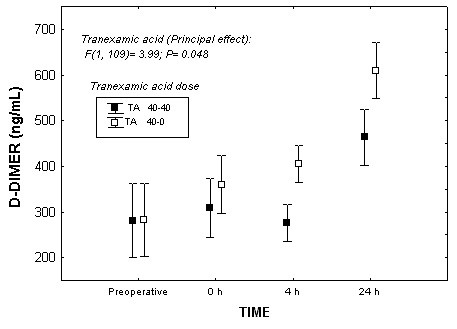
**Mixed ANOVA showing the relationship between Tranexamic acid doses and D-Dimer during the follow up**.

In the omnibus test TA2 had significantly lower chest-tube bleeding (P = 0.014). Comparing both groups for chest bleeding at 24 hours, TA2 had lower bleeding than TA1 [978 (95% CI: 809-1147) vs 1198 (95%CI: 1017-1380)mL; *P *= 0.010, corrected-*P *significant], which remained significant after adjusting for surgery-related bleeding (*P *= 0.014, corrected-*P *significant). On summing intraoperative and 24 h postoperative bleeding, the effect of TA2 was significant: [1341 (95% CI: 1197-1486) vs 1538 (95%CI: 1391-1686) mL; *P *= 0.016, corrected-*P *significant]. Also in the omnibus test, TA2 had significantly lower D-dimer (*P *= 0.038) than TA1 (Figure 3). TA2 had lower D-dimer levels at 24 hours [489 (95% CI: 437-540) vs. 621(95% CI: 563-679) ng/mL; *P *= 0.01, corrected-*P *significant].

TA2 showed significant reduction of maximum CPK values [407(95%CI: 259-671) vs 487(95%CI: 359-812)U/L; *P *= 0.01] and TnI [2.6(95%CI: 1.7-4.7) vs 3.6(95%CI: 2.7-6.3)U/L; *P *> 0.01]. TA2 required lower levels of norepinephrine at 24 h [0.06 (95%CI: 0.03-0.09) vs 0.20 (95CI5%:0.05-0.35), *P *= 0.03, adjusted for dobutamine [F(1.40) = 6.6; *P *= 0.014, corrected-*P *significant].

No differences were found in hemoderivative requirements or other parameters (Table 2).

**Table 2:** Outcomes of treatment groups.

	Tranexamic acid		*P*
	TA1 group (40-0 mg/Kg) (n = 80)	TA2 group (40-40 mg/Kg) (n = 80)	
D-dimer (ng/mL) 0-h	363 (307-420)	311 (257-365)	0.038*
D-dimer (ng/mL) 4-h	416 (376-455)	283 (254-312)	
D-dimer (ng/mL) 24-h^a^	621 (563-679)	489 (437-540)	
Chest tube bleeding (cc) 0 h	136 (109-163)	109 (89-129)	0.014†
Chest tube bleeding (cc) 4 h	366 (282-449)	263 (222-304)	
Chest tube bleeding (cc) 24 h^b^	826 (704-949)	671 (549-793)	
Chest tube total bleeding (cc)	1198 (1017-1380)	978 (809-1147)	
Transfused patients at 24 h (%)	22 (27.8)	19 (23.8)	0.55
Inflammatory response (%)	15 (18.8)	6 (7.5)	0.03
Temperature > 38 (°C) n(%)	15(18.8)	6(7.5)	0.03
Cardiac index > 3.5 (L/min/m^2^) n(%)	65(82.3)	58(75.3)	0.29
Systemic vascular resistance index <1600 (dyne · sec/cm^5 ^per m^2^) n(%)	31(39.2)	21(27.6)	0.13
Interleukine-6 (pg/mL) 0-h	281 (139-423)	239 (177-302)	0.79‡
Interleukine-6 (pg/mL) 4-h	511 (341-679)	470 (327-613)	
Creatine kinase (U/L) 0 h	257 (207-352)	208 (160-315)	0.12¶
Creatine kinase (U/L) 4 h	336 (253-460)	281 (228-414)	
Creatine kinase (U/L) 24 h	447 (336-807)	399 (266-657)	
Creatine kinase peak (U/L)	487 (359-812)	407 (259-671)	0.01
Troponin I (ng/mL) 0 h	1.3 (1-2.8)	1.2 (0.6-1.7)	0.88**
Troponin I (ng/mL) 4 h	3.4 (2.4-5.3)	2.3 (1.6-3.9)	
Troponin I (ng/mL) 24 h	1.8 (1.2-3.3)	1.6 (1.1-3.3)	
Troponin I peak (ng/mL)	3.6 (2.7-6.3)	2.6 (1.7-4.7)	<0.01
Norepinephrine (mcg/Kg/min) 0 h	0.07(0.03-0.12)	0.06(0.02-0.10)	0.46^††^
Norepinephrine (mcg/Kg/min) 4 h	0.12(0.04-0.19)	0.09(0.03-0.14)	
Norepinephrine (mcg/Kg/min) 24 h	0.20(0.05-0.35)	0.06(0.03-0.09)	
Dobutamine (mcg/Kg/min) 0 h	1.77(1.03-2.51)	1.68(0.99-2.38)	0.96^‡‡^
Dobutamine (mcg/Kg/min) 4 h	1.60(0.80-2.41)	1.69(0.94-2.44)	
Dobutamine (mcg/Kg/min) 24 h	1.19(0.47-1.92)	1.28(0.59-1.98)	
Mechanical ventilation (h)^d^	7 (5-13)	7 (6-15)	0.60
Re-intervention, n (%)	5 (6.3)	2 (2.5)	0.44
Postoperative stroke, n (%)	1 (1.3)	2 (2.5)	0.56
Seizures, n (%)	0 (0)	2 (2.5)	0.49
Renal dysfunction, n (%)	6 (7.5)	7 (8.9)	0.75
Renal failure, n (%)	4 (5)	3 (3.8)	0.70
Myocardial infarction, n (%)	2 (2.5)	1 (1.3)	0.56
Intensive care length of stay (days)^c^	3 (2-5)	3 (2-5)	0.26
Mortality, n (%)	3 (3.8)	6 (7.6)	0.29
Composite Adverse Effects(%)	14(17.5)	11(13.8)	0.51

Values expressed as means and 95% confidence interval; frequencies and percentages.

^a^D-dimer levels at 24 hours (*P *= 0.01, corrected *P *significant). ^b ^Chest bleeding at 24 hours (*P *= 0.01, corrected *P *significant). ^c^Norepinephrine at 24 h adjusted for dobutamine at 24 h (P = 0.014, corrected *P *significant). ^d^Values expressed as median and interquartile range

* † ‡ ¶ ^** †† ‡‡ ^*P *values correspond to omnibus F-scores and were obtained using Mixed ANOVA

We found a direct relationship between IL-6 at ICU arrival and: temperature (rho = 0.26; *P *< 0.01), D-dimer (rho = 0.24; *P *< 0.01), norepinephrine (rho = 0.33; *P *< 0.01), TnI (rho = 0.37; *P *< 0.01), CK(rho = 0.37; *P *< 0.01), CK-MB(rho = 0.33; *P *< 0.01) and lactic acid (rho = 0.46; *P *< 0.01) at arrival. Similarly, a direct correlation was observed between IL-6 at 4 h with temperature (rho = 0.23; *P *< 0.01), norepinephrine (rho = 0.25; *P *< 0.01), TnI (rho = 0.42; *P *< 0.01) and lactic acid (rho = 0.19; *P *= 0.03) at 4 h and chest tube bleeding at 24 h (rho = 0.18; *P *= 0.03) and at chest-tube withdrawal (rho = 0.20; *P *= 0.02), and an inverse relationship with systolic blood pressure at 4 h (rho = -0.17; *P *= 0.05).

Seizure was present in 2 (1.3%) and stroke in 3 (1.9%) patients. Acute kidney injury occurred in 14 (8.8%) patients, and 7(4.4%) needed dialysis. Finally, 3 (1.9%) suffered myocardial infarction. Seven patients required re-operation for bleeding: 5 in TA1 and 2 in TA2,(*P = 0.44*). There were 9 deaths, without significant differences between groups: 3 (3.8%) in TA1 versus 6 (7.6%) in TA2 (*P *= 0.29). Statistically, there were no significant differences in expected vs. observed mortality between the two groups. According to logistic Euroscore, the rates of mortality were: TA1-observed = 3.8% (3/80) and TA1-expected = 3.25% (3/80), (*P *= 0.99) and TA2-observed = 7.6% (6/80)] and TA2-expected = 3.27% (3/80), (*P *= 0.11). There were no significant differences between groups in cerebrovascular events, renal pathology, myocardial infarction, mechanical ventilation or ICU stay after surgery, nor when comparing composite adverse effects (Table 2).

## Discussion

The clinical definition of IR based primarily on early hyperthermia with a hyperdynamic state is accompanied by higher levels of IL-6 at 4 h than in other patients. Peak levels of this interleukin is observed in these first hours of the postoperative period, as found by our team in a previous study [6] and by others [16,17]. The presence of hyperthermia in the criteria used avoids the potential confounding effect of vasoactive drugs on the associated hyperdynamic state.

Our findings indicate that prolonged inhibition of fibrinolysis by a postoperative dose of TA reduces IR and bleeding in CPB patients. TA1 patients presented higher levels of D-dimer, greater bleeding and IR than TA2 patients who received a second dose after CPB.

Several mechanisms have been proposed to explain IR after CPB, including contact activation, ischemia-reperfusion, and endotoxemia. These triggers may activate numerous systems involving complement, cytokines, immune cellular response and coagulation-fibrinolytic cascades [4]. These systems are closely interconnected and provide continuous feedback, so the release of cytokines[18] or activation of the complement system [19] may amplify the fibrinolytic response. This in turn may re-activate the release of inflammatory mediators by plasmin and D-dimer [3,20,21]. This amplified IR is especially relevant in CPB.

In a previous study, we observed significantly higher levels of IL-6 in patients with IR and TA was effective in reducing this response [6]. Thus in the present study we focused on IL-6 and fibrinolysis. In addition to the feed-back between the release of inflammatory markers and fibrinolysis, other components must be considered in the release of these markers. After CPB (Figure 2) we observed a clear increase in the levels of IL-6 and other interleukins, which interact with each other [16,17] and are involved in producing fever ("postperfusion syndrome") [22]. Also, oxidative stress induced by norepinephrine may release IL-6 in other settings [23]. In this study, we observed no differences in IL-6 levels between groups, probably because both them received antifibrinolytic.

Proinflammatory cytokines may contribute to inflammatory response, myocardial ischemia-reperfusión injury and hemodynamic instability after clinical CPB. This myocardial dysfunction has been highlighted in diverse studies through the association between cytokine levels (IL-6) and markers of myocardial tissue damage such as TnI y CK [24,25]

In the present study, patients developing IR showed higher levels of IL-6 than those who did not, together with worse hemodynamic status. The association between greater cytokine (IL-6) release, activation of fibrinolysis (D-dimer), higher temperature, higher levels of TnI, CK, CK-MB, and increased hemodynamic instability (norepinephrine requirements and higher lactate levels) in the immediate postoperative period support the inter-relation between inflammation and fibrinolysis [18-20].

The protective effect of TA regarding IR, more evident in the TA2 group, was also reflected in less postoperative myocardial tissue damage.

Optimal use of inotropes or vasopressors in the perioperative period of cardiac surgery remains controversial. We usually employ a combination of dobutamine plus norepinephrine in patients who develop postoperative cardiac dysfunction when there is associated hypotension, to maintain an adequate perfusion pressure. This would justify the use of norepinephrine in patients who do not develop IR [26,27]. Although the initial analysis revealed no differences in the need for norepinephrine in the first 24 hours postoperatively between the treatment groups, differences did appear at 24 h after adjusting for the dose of dobutamine at the time.

Although TA effectively reduces bleeding after cardiac surgery, doses vary widely [12,13]. Most are based on an initial dose and subsequent infusion, which can result in high cumulative doses. TA half-life is 80 min, and the effect of a single dose (TA1) is nearly 100% inhibition of plasmin activity[14] in the immediate postoperative period, so a second dose administered at the end of surgery may prevent IR and bleeding complications, since enhanced fibrinolysis might be expected after heparin reversal [28].

Thus the dosing schedule used in our study ensures complete inhibition of fibrinolysis, although no pharmacokinetic information was available. With the second dose we wished to investigate the possible benefit of postoperative inhibition of fibrinolysis on IR.

It is questionable whether the statistically smaller blood loss in TA2 group is really clinically relevant, in terms of transfusion requirements. The use of hemoderivatives partly depends on the transfusion policy of each center, as in our case. However, given that numerous authors concur on the need for antifibrinolytic agents in cardiac surgery [29], we believe the protective effect of the TA2 dosing schedule used in the present study on IR and clinical parameters should be considered.

The incidence of non-ischemic clinical seizure varies from 2.7% to 4.6% in most major surgical procedures [8-10], only 0.4% in CABG [9], but as high as 6.7% [8] or 7.9% [9] in open chamber surgery. This adverse effect has been reported with different several dosing schedules and cumulative doses of 61-259 mg/kg [8-10].

The incidence of non-ischemic clinical seizure was 0% in the single-dose group versus 2.5% in the double-dose group (non-significant difference); one patient underwent CABG and the other open chamber surgery (valve replacement), and both had associated renal dysfunction. Although our incidence was below the limits reported by other authors, with slightly higher cumulative doses than those used by Martin K et al [9] and lower than Sander M et al [8] and Murkin JM et al [10], there seems to be a certain dose-dependent relationship with seizure.

Tranexamic acid has been shown to have an epileptogenic effect in animals [30]. The suspected mechanism is a γ-aminobutiric acid-driven inhibition of the central nervous system [31]. The patho-mechanism of the two seizures recorded in our study remains undetermined. Both occurred in patients with postoperative renal dysfunction which facilitates accumulation of the drug, and one patient underwent aortic valve surgery where micro-plaque dislodgement or the presence of micobbubles, which inevitably remain after closing the heart, could have triggered the seizure. This may also partly explain the higher incidence of seizure in open chamber surgery

Cerebrovascular events, renal pathology and myocardial infarction were similar to those reported previously[32]. Hospitalization and mortality were similar in both groups.

## Limitations

With 80 patients in each group, we empirically estimated a 13% difference between the incidence rates of IR, because in the balance between safety and effectiveness, we preferred safety. Although the empirical power for the observed proportions (18.8 vs. 7.5) was 67%, the study was sufficiently powered and significant differences were found at the end of the study.

Although the incidence of adverse events observed are consistent with that reported in other series, the study was underpowered to be able to affirm that the dosing schedule was safe since the sample size was calculated for IR.

Only IL-6 was measured as a biochemical marker of inflammatory response to CPB. However, this cytokine is widely used in this context, as shown in the literature [16,17].

Blood levels of TA were not measured, but the pharmacokinetic profile of this drug has been previously studied by other authours and the dosing schedule was based on their findings [14].

Lastly, the experimental nature of this single-center study with CPB patients undergoing a particular surgical technique (with non-coated circuits), limits the external validity of our findings.

## Conclusions

In conclusion, an additional postoperative dose of TA may reduce IR and postoperative bleeding (but not transfusion requirements) in CPB patients. A question which remains unanswered is whether the dose used was ideal in terms of safety, but in terms of effectiveness we have no doubt

## List of abbreviations

CPB: Cardiopulmonary bypass; TA: Tranexamic acid; IR: Inflammatory response; MOF: Multiorganic organ failure; ICU: Intensive care unit; PT: Prothrombin time; IL-6: Interleukin-6

## Competing interests

The authors declare that they have no competing interests.

## Authors' contributions

JJJ and JLI were responsible for the study design, data collection, processing blood samples during the study, statistical analysis, data interpretation, and drafting the manuscript. DH, AJ were responsible for the statistical analysis, data interpretation, and drafting the manuscript. MB, LL, RP and MLM, were responsible for data collection and processing blood simples during the study and provided useful suggestions. PM and JMR, was responsible for determination of coagulation-fibrinolysis parameters and interpretation. JMB and BM, was responsible for determination of IL-6 and interpretation. RM was the surgical team and was responsible for preoperative clinical and analytical data collection. All authors read and approved the final manuscript.
